# Adherence to therapeutic recommendation in relapsing-remitting multiple sclerosis patients

**DOI:** 10.3389/fimmu.2025.1545430

**Published:** 2025-06-26

**Authors:** Justyna Chojdak-Łukasiewicz, Aleksandra Kołtuniuk, Ewa Sawicka, Anna Pokryszko-Dragan

**Affiliations:** ^1^ Department of Neurology, Wroclaw Medical University, Wrocław, Poland; ^2^ Department of Nursing, Wroclaw Medical University, Wrocław, Poland; ^3^ Department of Toxicology, Wroclaw Medical University, Wrocław, Poland

**Keywords:** multiple sclerosis, relapsing-remitting multiple sclerosis, adherence, non- adherence, acceptance of illness

## Abstract

**Background:**

Adherence to disease-modifying therapies (DMTs) in multiple sclerosis (MS) is crucial for effective treatment. Understanding the patients’ perspective and identification of factors influencing adherence to treatment may allow improved disease management.

**Objective:**

This study attempted to evaluate adherence to DMTs in people with MS and explore potential patient-related factors influencing level of adherence.

**Methods:**

121 patients with relapsing-remitting MS (90% women, aged 34.0 ± 10 years participated in the online self-reported survey, which included: Adherence in Chronic Diseases scale (ACDS), Patient health questionnaire (PHQ-9) as a measure of depression, Modified Fatigue Impact Scale (MFSI), Perceived Stress Scale (PSS-10), Acceptance of Illness Scale (AIS), and a questionnaire about demographics and basic patient-reported clinical data.

**Results:**

The ACDS results comprised between 9 and 28 points, with a mean value of 24.0 ± 3.8, indicating a moderate level of adherence. Fatigue was reported by 70% of patients and the results of MFIS showed significant correlation with ACDS (R=-0.260; p=0.004) and AIS score (R=0.487; p <0.001). Relationships were found between the results of AIS and PHQ-9 and PSS-10 but none of them was associated with ACDS, and neither were demographic data.

**Conclusions:**

The adherence to treatment among MS patients is moderate and significantly associated with fatigue. Evaluating the level of adherence and the patients’ psychosocial condition may contribute to personalized and optimal therapeutic approach.

## 1 Introduction

Multiple sclerosis (MS) is a chronic, autoimmune demyelinating disease of the central nervous system (CNS), affecting about 2.8 million people worldwide (1, 2). The pathogenesis of MS is complex and still not completely elucidated; it involves immune-mediated inflammatory response and neurodegenerative component with axonal loss. Multifocal symptoms can vary depending on the affected areas of the CNS. MS is a long-term condition characterized by dynamically changing, hardly predictable course. The most common type of MS is relapsing- remitting MS (RRMS) characterized by clearly defined attacks of new or recurrent neurologic symptoms, followed by periods of partial or complete recovery (remissions). Approximately 20% of patients with RRMS convert with time to secondary progressive MS (SPMS) and ca. 15% presents with primary progressive MS (PPMS), characterized by a gradual worsening of neurological deficit from the disease onset. Currently recommended therapeutic approach in MS is complex and includes treatment of acute relapse, disease-modifying therapies (DMTs) and symptomatic treatment, supported with management of comorbidities, psychological counselling, rehabilitation regimens and lifestyle modifications.

Due to the recent substantial progress in MS research, several DMT are currently available (with various mechanisms of action, routes of entry, efficacy, and side-effect profiles), aimed at effective control of the disease activity and prevention of its consequences. The primary goal of DMT is defined as NEDA (“no evidence of disease activity”) status, characterized by a lack of relapses and/or progression of disability and/or new lesions in subsequent magnetic resonance imaging (MRI) of the CNS (3). Moreover, currently expected outcomes of treatment include improvement in various aspects of disability as well as overall quality of life and functioning in its particular spheres (e.g. vocational activity, social participation, family planning) (4).

Similarly to variety of symptoms and dynamics of the MS course in the population of patients, significant individual differences are also observed in the response to DMT. They may be associated with disease-related, drug-related, or patient-related factors. Among the latter, adherence to treatment and communication with healthcare providers are essential for effective management of treatment strategy.

According to the World Health Organization (WHO), adherence is defined as “the extent to which patients follow medical instructions.” Adherence is particularly important for effective treatment of chronic conditions. In MS non-adherence to DMT has been shown to contribute to adverse clinical (unfavorable course - higher relapse rate, greater increase in disability) and socioeconomic effects (frequency of MS-related hospitalizations and absences from work, increased costs of healthcare). Understanding the MS patients’ perspective and identification of factors influencing their adherence to treatment seems crucial for adapting personalized approach to therapy and achieving its optimal effectiveness (5).

The purpose of the study was to assess the degree of adherence to treatment with DMT in the patients with MS and to identify patient-related factors potentially affecting the adherence, including demographics, patients’ perspective of disease burden and psychosocial issues (depression, fatigue, exposure to stress).

## 2 Materials and methods

### 2.1 Study design

The design of the study was an online survey-based, cross-sectional research. The link was distributed to adults’ patients belonging diagnosed with relapsing-remitting multiple sclerosis (RRMS), being under regular follow-up at the Department of Neurology, Wroclaw Medical University, Poland. Treatment of MS in Poland, due to reimbursement regulations of healthcare authorities, is organized within so-called therapeutic programme. The patients diagnosed with MS are qualified for DMTs and continue their treatment under the charge of regional specialist centers. Regular follow-up visits include monitoring of DMT efficacy and safety, evaluation of other drug and disease-related issues (including adherence) and providing the patients with adequate supply of medication until the next visit. On the basis of this follow-up, decisions are made about continuation or switch of DMT and other therapeutic aspects. Originally the main goal of this programme was to control the costs of treatment and availability of DMT was limited. However, in a few recent years an access to DMTs and indications for their use in the programme have been made mostly consistent with up-to-date recommendations (6). Moreover, the uniform regimen of the patients’ follow-up in the centers throughout the country allows better quality of MS management and regular care for the patients.

Data collection was conducted between November 2023 and January 2024. Before participants could proceed with the questionnaires, they were required to thoroughly review and sign a comprehensive consent document. This form provided a full explanation of the study’s objectives, potential risks and advantages, and outlined the measures in place to protect the privacy and confidentiality of their personal information.

The inclusion criteria were:

1. age>18 years old.
2. confirmed diagnosis of RRMS, according to the revised McDonald criteria for 2017 (7).
3. treatment with DMT scheduled and continued for at least one year.
4. consent for participation in the study.

The exclusion criteria included:

1. progressive types of MS.
2. starting or switching DMTs within the preceding year.
3. severe cognitive impairment (limiting the possibility to fill in self-report online questionnaires).

### 2.2 Instruments

#### 2.2.1 Data collection instruments

The questionnaire designed by the authors included questions about sociodemographic data: age, gender, place of living, level of education, employment status, marital status) and disease traits (duration of the disease, most bothering symptoms; frequency of relapses and the treatment used).

#### 2.2.2 Self-reported questionnaires

1. The adherence scale in chronic diseases (ACDS) is a standardized self-reported questionnaire to gauge the level of adherence to treatment in chronic diseases. The questionnaire contains seven questions: the first five relate to the patient’s behavior towards the medication; questions 6 and 7-to situations and opinions that affect adherence. For each question one answer should be chosen from 5 predefined options scored on a 4-point scale. The final score >26 points indicate high adherence; scores of 21-26-intermediate and below 21 points-poor adherence (8).
2. The Patient’s Health Questionnaire (PHQ-9) is a multipurpose self-reported test for measuring the degree of depression. The questionnaire comprises nine statements, with corresponding options to choose rated on a 4-point scale ranging from 0 (not at all) to 3 (nearly every day). The total score is 27 points. The result can be classified as ‘‘ severe depression’’ (20–27 points),moderate depression’’ (15–19 points),mild depression ‘‘ (10–14 points), and no depression’’ (9 or fewer points) (9).
3. Modified Fatigue Impact Scale (MFSI) is a tool dedicated to evaluating the level of fatigue. 21 items check how often fatigue has impacted particular functions during the past 4 weeks using a 5-point scale (0-never;4-almost always). The MFSI can be divided into three subscales: cognitive (10 items), physical (9 items), and psychosocial fatigue (2 items) (10).
4. Perceived stress scale (PSS-10) is a self-reported test assessing the global perception of stress. It consists of answers to 10 questions, rated on a 5-point scale (0-never, 4-very often). The total score of PSS-10 is 40. Scores ranging from 0–13 indicate low level, 14-26 - moderate level and 27-40 - high level of perceived stress (11).
5. The Acceptance of Illness Scale (AIS) is a self-reported questionnaire applied to evaluate patients’ attitude towards the disease. It contains 8 statements, with responses graded on a scale from 1 to 5. The total maximum score of AIS is 40. Scores below 20 points correspond with low level, between 21 and 30 points – with moderate and above 30 points – with high level of illness acceptance (12).

The data were automatically transferred to a spreadsheet, streamlining the data collection process and reducing the potential for manual input errors. The data were anonymized to protect participant privacy and ensure confidentiality during the research process.

### 2.3 Ethical considerations

The study was approved by the Bioethics Committee of Wroclaw Medical University. Participation in the research was entirely voluntary, with participants’ identities protected through complete anonymity. Prior to commencing the study, researchers obtained written informed consent from each patient, ensuring ethical standards were upheld.

### 2.4 Statistical analyses

All statistical analyses were performed using STATA 18, Stata Statistical Software: Release 18. StataCorp LP, College Station, TX, USA.

The results of all the questionnaires involved in the survey were subjected to descriptive statistical analysis. Relationships were searched between the results of ACDS and all the other data obtained from the responses to the questionnaires.

Continuous variables were expressed in the format of a mean with a standard deviation. Categorical variables were presented as a combination of numbers and percentages. When the data distribution was normal, the Student t-test was used to compare means of corresponding variables between groups. The normality of the distribution of various continuous variables was evaluated with a Shapiro–Wilk test. Spearman correlation was applied, to correlate variables. Results were determined to be statistically significant when p-value < 0.05.

## 3 Results

A total of 121 patients with RRMS (90% female ones) with median age 34.0 years (range: 18 to 58 years) completed the survey. The demographics and clinical characteristics are shown in **Table 1**.

**Table 1:** Demographic and clinical characteristics of the study group (n=121).

Variables	Value n (%)
Gender woman men	109 (90%) 12 (10%)
Place of residence Village City <20 thousand residents City>20 thousand residents City > 50 thousand residents City >100 thousand residents	32 (26%) 11 (9%) 11 (9%) 11 (9%) 56 (46%)
Education basic or vocational education secondary education higher education	1 (1%) 41 (34%) 79 (65%)
Marital status single married divorces	54 (45%) 57 (47%) 10 (8%)
Occupational activity active/working retired pensioner unemployed student	12 (10%) 89 (74%) 15 (12%) 4 (3%) 1 (1%)
Duration of the disease <2 yrs 2-5 yrs 6-10 yrs > 10 yrs	47 (39%) 40 (33%) 21 (17%) 13 (11%)
Frequency of relapses (per year) 0 1 > 1	49 (40%) 54 (45%) 18 (15%)

The results of ACDS ranged between 9 and 28 points, the mean value was 24 ± 3.8. In the majority (62%) of the patients ACDS result showed a moderate level of adherence to the treatment (**Table 2**).

**Table 2:** Results of the ACDS scale.

ACDS-score	Interpretation	n (%)
0-20	low level of adherence	14 (12 %)
21-26	moderate level of adherence	75 (62 %)
27-28	high level of adherence	32 (26 %)

ACDS Adherence in Chronic Diseases Scale, n- number of patients.

Fatigue and mood disturbances were most reported by respondents to the survey as the most bothering symptoms of MS. The same referred to subgroup of patients with high level of adherence, while in those with moderate level fatigue was followed by visual deficit, and in those with low adherence – by sensory disturbances (**Table 3**).

**Table 3:** The most bothering symptoms of MS reported by the patients in the study group.

Variables	all patients n=121	High level of adherence n=32	Moderate level of adherence n=75	Low level of adherence n=14
fatigue	85 (70%)	19 (59%)	55 (73%)	11 (79%)
mood disturbances	65 (54%)	16 (50%)	41 (55%)	4 (29%)
sensory disturbances	58 (48%)	14 (44%)	36 (48%)	8 (57%)
balance problem	49 (41%)	7 (22%)	25 (33%)	6 (43%)
visual disturbances	30 (25%)	8 (25%)	19 (63%)	3 (21%)
pain	13 (11%)	6 (19%)	5 (7%)	2 (14%)

**Figure 1:**
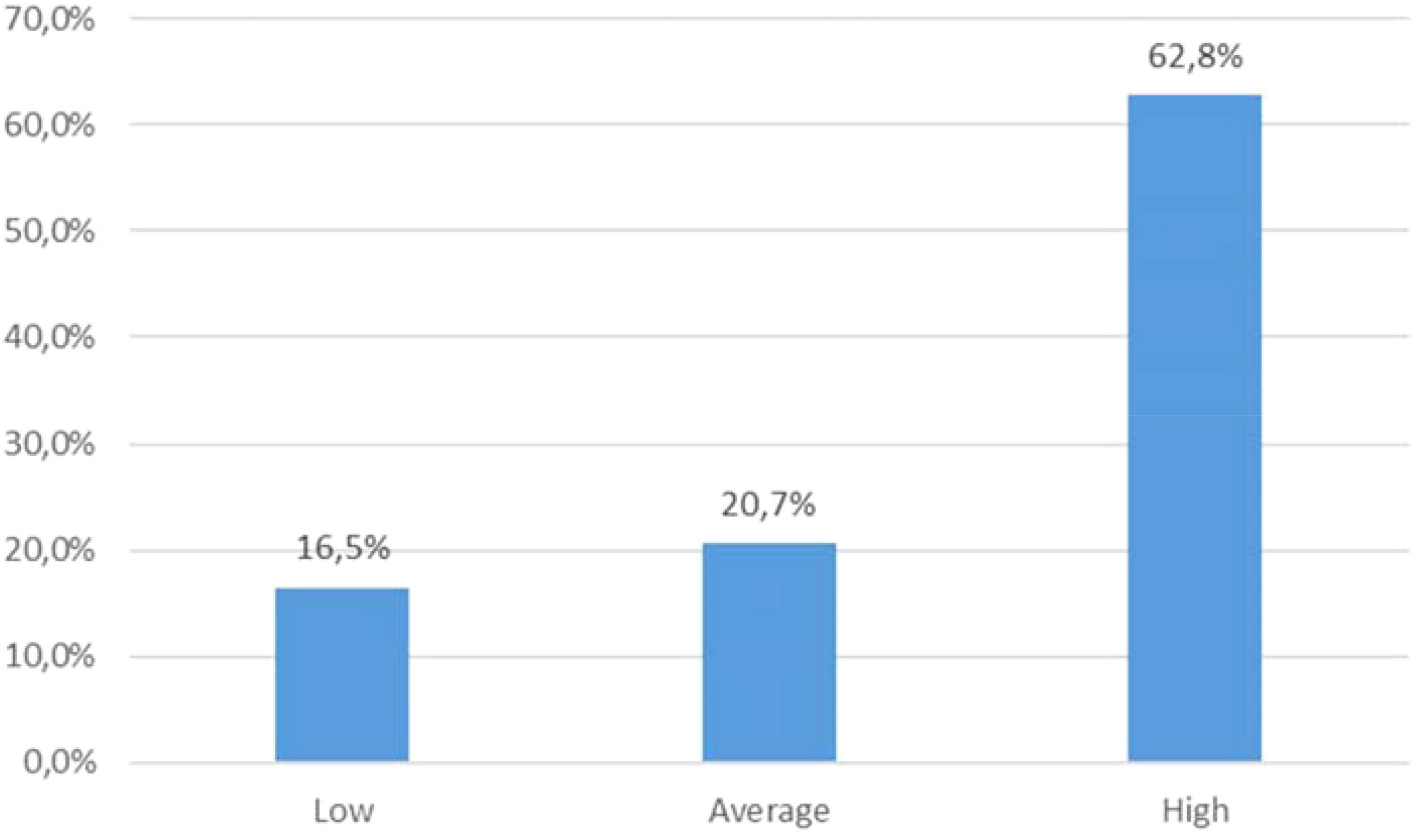
Distribution of PSS-10 results (sten scale) in the study group.

**Figure 2:**
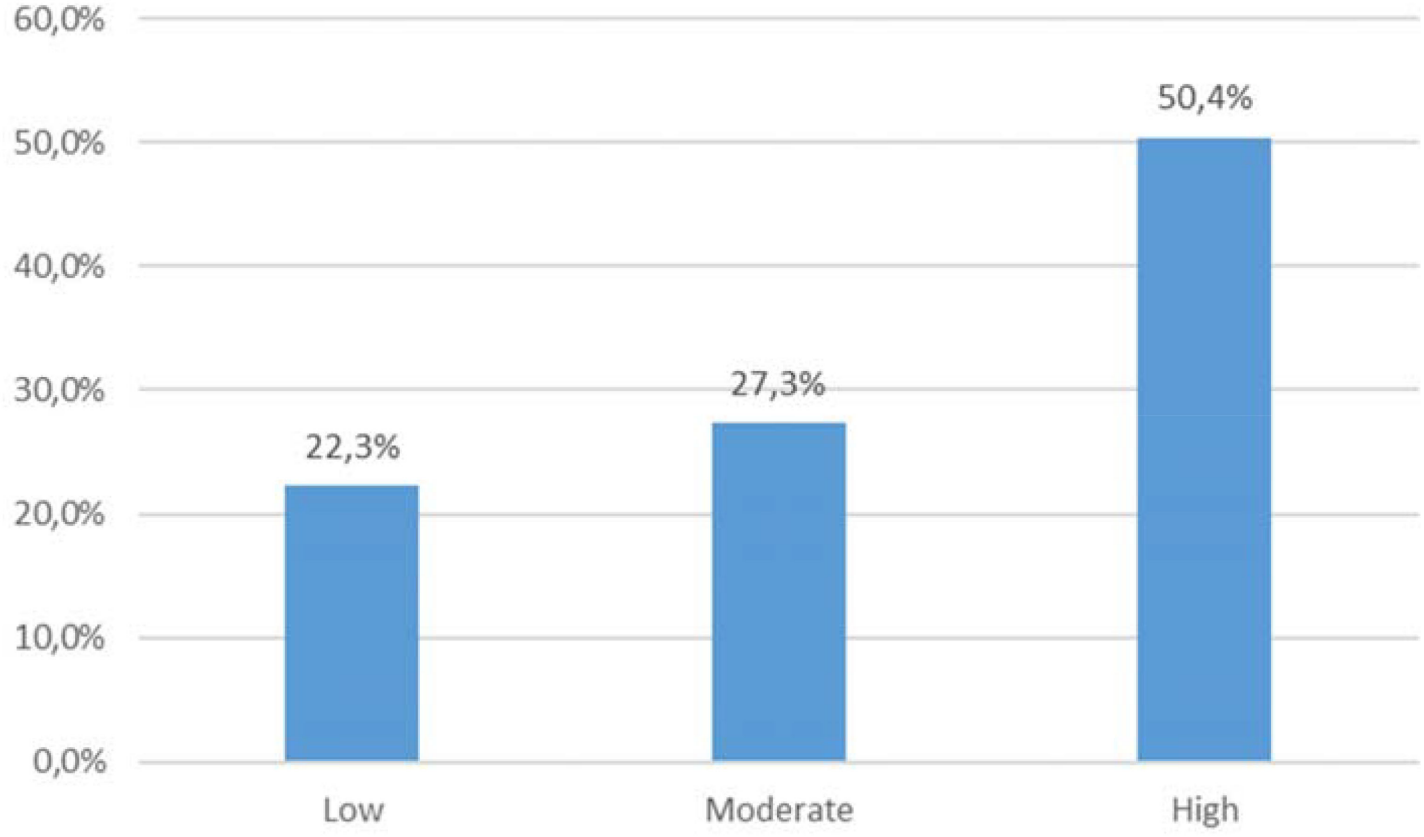
Acceptance of multiple sclerosis according to AIS (Acceptance of Illness Scale).

The mean result of the MFSI scale for the study group was 37.5 ± 19.3. The average PSS-10 score was 21.83 (range: 0-36). According to the sten scale, 20 individuals (16,5%) declared low level of perceived stress, 25 (20.7%) - moderate, and 76 (62.8%) - high. (**Figure 1**). The mean score of PHQ-9 was 12.9 ± 8.1 (**Table 4**). Based on the results, 59 (49%) patients presented with no depression, and 62 (51%) reported depressive symptoms: among these, 38 patients were assigned as mildly depressed, 15 - moderately, and 9 - severely depressed. According to AIS results, the average score was 28.2 ± 9.5, which is consistent with moderate level of illness acceptance (**Figure 2**).

**Table 4:** Summary of results of questionnaires in the study group.

Variables	Study group n=121 mean value ±SD (min-max)
PHQ9	12.9 ±8.1 (0-25)
AIS	28.2±9.5/(8-40)
ACDS PSS-10	24.0±3.8 (9-28) 21.83 ± 7.6 (0-36)
MFIS-total	37.5±19,3 (1-83)
MFIS-Phy	17.5±8.7 (0-36)
MFIS-Cog	16.2±10 (0-40)
MFIS-Pso	3.8±2.4 (0-8)

PHQ9-; AIS-; ACDS MFSI-total Modified Fatigue Inventory Scale; MFIS-Phy, Modified Fatigue Inventory Scale-Physical; MFIS-Cog, Modified Fatigue Inventory Scale-Cognitive; MFIS-Pso, Modified Fatigue Inventory Scale-Psychosocial.

On analysis of relationships between ACDS results and other parameters, the only significant negative correlation was found for the MFIS (total score and all subscores) (**Table 5**), indicating that fatigue in all dimensions (physical, cognitive, and psychosocial) was related to poorer adherence. No significant associations were revealed between ACDS results and demographics or MS- related variables. Furthermore, significant associations were found between AIS score and the results of MFIS, PHQ9 and PSS-10 (**Table 6**), illustrating that depression, level of perceived stress and fatigue are related to lower illness acceptance. We also found the correlation between PHQ-9 and fatigue scale scores (rho 0.634; p<0.001).

**Table 5:** Correlations between ACDS score and clinical variables in the study group.

Variables	ACDS score	
	Rho	P
PHQ9	-0.173	0.06
AIS	0.149	0.10
PSS-10	-0.016	0.86
MFIS-total	-0.260	**0.004**
MFIS-Phy	-0.326	**<0.001**
MFIS-Cog	-0.186	**0.041**
MFIS-Pso	-0.183	**0.044**
Age	-0.012	0.89
Duration of the disease	-0.108	0.24
Frequency of relapses	-0.109	0.23

Spearman's ρ(Ro) -nonparametric correlation.Bold values indicates p<0.05 statistical significant.

**Table 6:** Correlations between AIS score and clinical variables in the study group.

Variables	AIS score	
	Rho	p
PHQ9	-0.502	**<0.001**
PSS-10	-0.352	**<0.001**
MFIS-total	0.487	**<0.001**
MFIS-Phy	0.569	**<0.001**
MFIS-Cog	0.349	**<0.001**
MFIS-Pso	0.540	**<0.001**
Age	-0.184	**0.044**
Duration of the disease	-0.178	0.05
Frequency of relapses	-0.132	0.15

Spearman's ρ(Ro) -nonparametric correlation.Bold values indicates p<0.05 statistical significant.

No other significant correlations were found between analyzed variables.

## 4 Discussion

The goal of our study was evaluating the level of adherence to disease-modifying treatment in MS patients and its relationship with potentially contributing factors. Our results indicate that patients with MS follow the therapeutic recommendations to a moderate degree. In the other studies estimated adherence to the DMTs in MS ranges widely between 41% and 93%, depending on the study sample or measurement methods (13–16). Olkiewicz et al. and Thomason et al. demonstrated a high level of adherence in the majority of evaluated patients with MS (17, 18). Conversely, Hansen et al. found that less than 40% of patients were adherent to therapeutic recommendations during a 24-month observation period (19). Erbay et al. also revealed a low level of adherence in their study (20). These varying findings highlight the individual differences and complexity of the problem of therapeutic adherence among MS patients.

There are numerous factors that possibly influence adherence to treatment in MS patients, including disease-related, drug-related and patient-related issues (16). Shorter duration of MS and stable condition without relapses were shown to be associated with higher level of adherence (21). Similar relationship was found for intravenous mode of DMT administration as opposed to oral intake or subcutaneous injections (22).

However, in our study we deliberately focused only on the demographics and patient- related factors based on their self-report (with a special regard to psychological issues). While MS-related variables and drug characteristics are usually thoroughly considered before choice of DMT in view of therapeutic adherence, patients’ perspective may be underestimated.

Similar to our previous study (23), we didn’t find any significant correlations between adherence and sociodemographic factors. The findings from other studies are partly contradictory, indicating higher or lower level of adherence in younger patients (21, 24) and in women with MS (24, 25). Lower level of education and having a stable (marital or non-formal) relationship have been also linked to higher adherence to treatment in these patients (21, 22).

Fatigue is one of the most debilitating symptoms in MS, profoundly affecting the patients’ functioning and their quality of life (26, 27). Indeed, fatigue was most frequently reported by our study group participants (71%) and this was supported by quantitative results of MFIS. Moreover, we found a significant positive correlation between measures of fatigue (general and in all domains) and level of adherence. Some authors showed similar correlation between fatigue and non-adherence to treatment (28, 29), while others did not confirm such a relationship (30–32). Fatigue may prevent undertaking various activities by the patients, supposedly including taking medications (especially according to regular/demanding schedule). Thus, relieving fatigue by appropriate interventions would contribute to the better adherence to treatment. Sensory symptoms were associated in 70% of cases with moderate and low adherence levels. Memory problems or other deficits in cognitive performance have been shown to affect adherence to treatment in MS (e.g. causing omitting the doses, irregular schedule) (18, 21).

The impact of this factor was difficult to assess in our study group, as the patients did not report subjective cognitive problems and we did not include any test for cognitive functions into the online survey, expecting low reliability of results. Mood disorders were reported as the second most common bothering symptom in our study group. Considering frequency and relevance of depression in MS patients, we added PHQ-9 questionnaire to the survey as a tool for its assessment (33, 34). The results showed that half of the study group presented with depressive symptoms, which is consistent with findings from previous studies (frequency of depression ranging from 17.6% up to 79%) (35, 36). Although depression and fatigue in MS need to be distinguished, they may also coexist and overlap with each other. Another important aspect of the patients’ mental condition is associated with exposure to stress. Among environmental factors contributing to MS background, stress has been recently paid increasing attention because of its suspected impact upon MS activity (37). In this study 63% of the participants declared high level of stress according to PSS results, which is similar to findings from earlier studies in Polish MS patients (38–40). It is worth considering that burden of stress may be associated with the disease itself but also with non-health-related events or conditions. In the study survey no questions were asked about specific sources of stress, but we undertook additional evaluation of the patients’ perception of disease with the use of AIS. According to its results, more than 70% of the study group declared moderate or high level of disease acceptance, which suggests that they were able to adapt to their situations and attempt to come to terms with the limitations associated with the disease. Similar results were obtained in the other Polish studies concerning this aspect of living with MS (39–43). These findings may be associated with Polish system of MS management within so-called therapeutic programs (DMTs are reimbursed within these programs, providing patients with a regular follow-up in specialist healthcare settings with monitoring of effectiveness and safety of treatment).Significant correlations were found in our study between AIS results and measures of stress, depression and fatigue, as well as age, which highlights the factors influencing the acceptance of disease. Our findings are consistent with those from the previous studies (39, 40). On the contrary - apart from fatigue, none of the other factors analyzed in this study showed relationship with level of adherence to treatment. Other authors revealed such relationships e.g. for depression (25, 44, 45). However, our results suggest that evaluating MS patients’ overall psychosocial condition may allow appropriate management of their problems in this field, hopefully resulting in better communication and cooperation between physicians and patients, which might also include improved adherence to treatment. This study has certain limitations, which firstly include online form of survey (potential bias - preferring those who have internet access and are used to digital platforms). Excluding patients with progressive forms of MS might have also affected the results. Although we deliberately focused on the subjective patient-reported data, the lack of objective MS-related variables (e.g. degree of disability or verified frequency of relapses) as well as the type of DMT used would probably provide better insight into problems of adherence. To mitigate these limitations and leverage the strengths of the study, future investigations are planned, combining self-reported survey with broader set of clinical data.

## 5 Conclusions

The findings from the study demonstrated moderate level of adherence to treatment in RRMS patients. Fatigue, reported as the most common bothering symptom of MS, showed significant inverse correlation with level of adherence. Demographics and patient-reported clinical data were not associated with adherence to treatment. Relationships were shown between level of disease acceptance and depression, perceived stress and fatigue. Evaluating MS patients’ adherence to treatment, as well as their psychosocial condition may contribute to personalized approach and improvement of overall disease management.

## Data Availability

The raw data supporting the conclusions of this article will be made available by the authors, without undue reservation.

## References

[1] McGinley MP Goldschmidt CH Rae-Grant AD. Diagnosis and treatment of multiple sclerosis: A review. JAMA. (2021) 325:765. doi: 10.1001/jama.2020.26858 33620411

[2] Walton C King R Rechtman L Kaye W Leray E Marrie RA. Rising prevalence of multiple sclerosis worldwide: Insights from the Atlas of MS, third edition. Mult Scler. (2020) 26:1816–21. doi: 10.1177/1352458520970841 PMC7720355 33174475

[3] Rotstein D Solomon JM Sormani MP Montalban X Ye XY Dababneh D. Association of NEDA-4 with no long-term disability progression in multiple sclerosis and comparison with NEDA-3: A systematic review and meta-analysis. Neurol Neuroimmunol Neuroinflamm. (2022) 9:e200032. doi: 10.1212/NXI.0000000000200032 36224046 PMC9558627

[4] Hauser SL Cree BAC. Treatment of multiple sclerosis: A review. Am J Med. (2020) 133:1380–1390.e2. doi: 10.1016/j.amjmed.2020.05.049 32682869 PMC7704606

[5] Sabaté E. Adherence to Long-Term Therapies: Evidence for Action. Geneva, Switzerland: World Health Organization (2003). Available online at: http://www.who.int/chronic_conditions/adherencerepor. 14562485

[6] Kułakowska A Mirowska-Guzel D Bartosik-Psujek H Brola W Stasiołek M Głąbiński A. Disease modyfifying therapy in multiple sclerosis recommendations of Multiple Sclerosis and Neuroimmunology Section of Polish Neurological Society. Neurol Neurochir Pol. (2024) 58:569–85. doi: 10.5603/pjnns.102356 39737584

[7] Thompson AJ Banwell BL Barkhof F Carroll WM Coetzee T Comi G. Diagnosis of multiple sclerosis: 2017 revisions of the McDonald criteria. Lancet Neurol. (2018) 17:162–173. doi: 10.1016/S1474-4422(17)30470-2 29275977

[8] Kubica A Kosobucka A Michalski P Pietrzykowski Ł Jurek A Wawrzyniak M. Skala adherence w chorobach przewlekłych — nowe narzędzie do badania realizacji planu terapeutycznego. Folia Cardiologica. (2017) 12:19–26. doi: 10.5603/FC.a2016.0105

[9] Kroenke K Spitzer RL Williams JBW. The PHQ-9: Validity of a brief depression severity measure. J Gen Intern Med. (2001) 16:606–13. doi: 10.1046/j.1525-1497.2001.016009606.x PMC1495268 11556941

[10] Larson RD. Psychometric properties of the modified fatigue impact scale. Int J MS Care. (2013) 15:15–20. doi: 10.7224/1537-2073.2012-019 24453758 PMC3883028

[11] Juczyński Z Ogińska-Bulik N. PSS 10. In: Narzędzia pomiaru stresu i radzenia sobie ze stresem. Pracownia Testów Psychologicznych, Warszawa (2009). p. 11–22.

[12] Juczyński Z. Skala akceptacji choroby - AIS. In: Narzędzia pomiaru w promocji i psychologii zdrowia. Pracownia Testów Psychologicznych Polskiego Towarzystwa Psychologicznego, Warszawa (2001). p. 168–72.

[13] Burks J Marshall T Ye X. Adherence to disease-modifying therapies and its impact on relapse, health resource utilization, and costs among patients with multiple sclerosis. CEOR. (2017) 9:251–60. doi: 10.2147/CEOR.S130334 PMC5417677 28496344

[14] Fischer MA Stedman MR Lii J Vogeli C Shrank WH Brookhart MA. Primary medication non-adherence: analysis of 195,930 electronic prescriptions. J Gen Intern Med. (2010) 25:284–90. doi: 10.1007/s11606-010-1253-9 PMC2842539 20131023

[15] Menzin J Caon C Nichols C White LA Friedman M Pill MW. Narrative review of the literature on adherence to disease-modifying therapies among patients with multiple sclerosis. JMCP. (2013) 19:S24–40. doi: 10.18553/jmcp.2013.19.s1.S24 PMC10437533 23383731

[16] Washington F Langdon D. Factors affecting adherence to disease-modifying therapies in multiple sclerosis: systematic review. J Neurol. (2021) 269(4):1861–72. doi: 10.1007/s00415-021-10850-w PMC8940867 34676448

[17] Olkiewicz J Bonek R Filipska K Ślusarz R. Adherence to therapeutic recommendations in patients suffering from multiple sclerosis. PNIN. (2020) 9:103–7. doi: 10.15225/PNN.2020.9.3.3 PMC8436112 34522188

[18] Thomason S Moghaddam N Evangelou N Middleton R das Nair R. Barriers and strategies to medication adherence amongst people with multiple sclerosis and cognitive problems. Multiple Sclerosis Related Disord. 88:105727. doi: 10.1016/j.msard.2024.105727 38905992

[19] Hansen K Schüssel K Kieble M Werning J Schulz M Friis R. Adherence to disease modifying drugs among patients with multiple sclerosis in Germany: A retrospective cohort study. PloS One. (2015) 10:e0133279. doi: 10.1371/journal.pone.0133279 26214805 PMC4516264

[20] Erbay Ö Usta Yeşilbalkan Ö Yüceyar N. Factors affecting the adherence to disease- modifying therapy in patients with multiple sclerosis. J Neurosci Nursing. (2018) 50:291–7. doi: 10.1097/JNN.0000000000000395 30138155

[21] Morillo Verdugo R Ramírez-Herráiz E Fernández-Del Olmo R Roig Bonet M Valdivia Garcia M. Adherence to disease-modifying treatments in patients with multiple sclerosis in Spain. PPA. (2019) 13:261–72. doi: 10.2147/PPA.S187983 PMC6388740 30863016

[22] Soria C Prieto L Lázaro E Ubeda A. Factors associated with therapeutic adherence in multiple sclerosis in Spain. PPA. (2023) 17:679–88. doi: 10.2147/PPA.S401962 PMC10024534 36941926

[23] Kołtuniuk A Chojdak-Łukasiewicz J. Strategies of coping with stress and the quality of life in relation to the adherence to therapeutic recommendations in MS — Affected patients. PNIN. (2021) 10:112–9. doi: 10.15225/PNN.2021.10.3.4

[24] Järvinen E Multanen J Atula S. Subcutaneous interferon β-1a administration by electronic auto-injector is associated with high adherence in patients with relapsing remitting multiple sclerosis in a real-life study. Neurol Int. (2017) 9(1):6957. doi: 10.4081/ni.2017.6957 28286627 PMC5337756

[25] Higuera L Carlin CS Anderson S. Adherence to disease-modifying therapies for multiple sclerosis. J Managed Care Specialty Pharmacy. (2016) 22:1394–401. doi: 10.18553/jmcp.2016.22.12.1394 PMC10397889 27882830

[26] Chalah MA Riachi N Ahdab R Créange A Lefaucheur JP Ayache SS. Fatigue in multiple sclerosis: neural correlates and the role of non-invasive brain stimulation. Front Cell Neurosci. (2015) 9:460. doi: 10.3389/fncel.2015.00460 26648845 PMC4663273

[27] Pinarello C Elmers J Inojosa H Beste C Ziemssen T. Management of multiple sclerosis fatigue in the digital age: from assessment to treatment. Front Neurosci. (2023) 17:1231321. doi: 10.3389/fnins.2023.1231321 37869507 PMC10585158

[28] Treadaway K Cutter G Salter A Lynch S Simsarian J Corboy J. Factors that influence adherence with disease-modifying therapy in MS. J Neurol. (2009) 256:568–76. doi: 10.1007/s00415-009-0096-y 19444532

[29] Tarrants M Oleen-Burkey M Castelli-Haley J Lage MJ. The impact of comorbid depression on adherence to therapy for multiple sclerosis. Multiple Sclerosis Int. (2011) 2011:1–10. doi: 10.1155/2011/271321 PMC3196992 22096632

[30] Von Gaudecker JR. Factors affecting the adherence to disease-modifying therapy in patients with multiple sclerosis. J Neurosci Nurs. (2018) 50:302–302. doi: 10.1097/JNN.0000000000000405 30198957

[31] Zanga G Drzewiscki E Tagliani P Smietniansky M Esnaola Y Rojas MM Caruso D. Predictors of adherence and persistence to disease-modifying therapies in Multiple Sclerosis. Ther Adv Neurol Disord. (2021) 14:175628642110310. doi: 10.1177/17562864211031099 PMC8495537 34630632

[32] Ślusarz R Olkiewicz J Bonek R Filipska K Biercewicz M Wiśniewski A. The impact of motor disability and the level of fatigue on adherence to therapeutic recommendations in patients with multiple sclerosis treated with immunomodulation. Int J Med Sci. (2021) 18:3609–14. doi: 10.7150/ijms.61964 PMC8436112 34522188

[33] Boeschoten RE Braamse AMJ Beekman ATF Cuijpers P van Oppen P Dekker J. Prevalence of depression and anxiety in Multiple Sclerosis: A systematic review and meta- analysis. J Neurological Sci. (2017) 372:331–41. doi: 10.1016/j.jns.2016.11.067 28017241

[34] Margoni M Preziosa P Rocca MA Filippi M. Depressive symptoms, anxiety and cognitive impairment: emerging evidence in multiple sclerosis. Transl Psychiatry. (2023) 13:264. doi: 10.1038/s41398-023-02555-7 37468462 PMC10356956

[35] Peres DS Rodrigues P Viero FT Frare JM Kudsi SQ Meira GM. Prevalence of depression and anxiety in the different clinical forms of multiple sclerosis and associations with disability: A systematic review and meta-analysis. Brain Behavior Immun - Health. (2022) 24:100484. doi: 10.1016/j.bbih.2022.100484 PMC9287158 35856061

[36] Hassan SS Darwish ES Ahmed GK Azmy SR Haridy NA. Relationship between disability and psychiatric outcome in multiple sclerosis patients and its determinants. Egypt J Neurol Psychiatry Neurosurg. (2023) 59:105. doi: 10.1186/s41983-023-00702-x

[37] Briones-Buixassa L Milà R Ma Aragonès J Bufill E Olaya B Arrufat FX. Stress and multiple sclerosis: A systematic review considering potential moderating and mediating factors and methods of assessing stress. Health Psychol Open. (2015) 2:205510291561227. doi: 10.1177/2055102915612271 PMC5193283 28070374

[38] Waliszewska - Prosół M Nowakowska - Kotas M Kotas R Bańkowski T Pokryszko - Dragan Podemski R. The relationship between event-related potentials, stress perception and personality type in patients with multiple sclerosis without cognitive impairment: A pilot study. Adv Clin Exp Med. (2018) 27:787–94. doi: 10.17219/acem/68944 29893512

[39] Kołtuniuk A Pytel A Kulik A Rosińczuk J. The role of disease acceptance, life satisfaction, and stress perception on the quality of life among patients with multiple sclerosis: A descriptive and correlational study. Rehabil Nurs. (2020) 46(4):205–13. doi: 10.1097/RNJ.0000000000000288 32932423

[40] Kołtuniuk A Kazimierska-Zając M Cisek K Chojdak-Łukasiewicz J. The role of stress perception and coping with stress and the quality of life among multiple sclerosis patients. PRBM. (2021) 14:805–15. doi: 10.2147/PRBM.S310664 PMC8219305 34177278

[41] Rosińczuk J Rychła K Bronowicka J Kołtuniuk A. The impact of ilness acceptance on quality of life of patients with multiple sclerosis — Preliminary study. PNIN. (2017) 6:157–62. doi: 46(4):205-213

[42] Pejas-Grzybek L Skorupska-Król A. The degree of illness acceptance among patients with multiple sclerosis. PNIN. (2015) 4:19–23. doi: 10.15225/PNN.2015.4.1.3

[43] Dymecka J Bidzan M. Biomedical variables and adaptation to disease and health-related Quality of life in Polish patients with MS. IJERPH. (2018) 15:2678. doi: 10.3390/ijerph15122678 30486508 PMC6313333

[44] Silveira C Guedes R Maia D Curral R Coelho R. Neuropsychiatric symptoms of multiple sclerosis: state of the art. Psychiatry Investig. (2019) 16:877–88. doi: 10.30773/pi.2019.0106 PMC6933139 31805761

[45] Munsell M Frean M Menzin J Phillips A. An evaluation of adherence in patients with multiple sclerosis newly initiating treatment with a self-injectable or an oral disease- modifying drug. PPA. (2016) 11:55–62. doi: 10.2147/PPA.S118107 28115831 PMC5221550

